# Analysis of postural control and muscular performance in young and elderly women in different age groups

**DOI:** 10.1590/bjpt-rbf.2014.0068

**Published:** 2015

**Authors:** Matheus M. Gomes, Júlia G. Reis, Regiane L. Carvalho, Erika H. Tanaka, Miguel A. Hyppolito, Daniela C. C. Abreu

**Affiliations:** 1 Departamento de Biomecânica, Medicina e Reabilitação do Aparelho Locomotor, Faculdade de Medicina de Ribeirão Preto (FMRP), Universidade de São Paulo (USP), Ribeirão Preto, SP, Brazil; 2 Escola de Educação Física e Esporte de Ribeirão Preto, USP, Ribeirão Preto, SP, Brazil; 3 Departmento de Oftalmologia, Otorrinolaringologia e Cirurgia da Cabeça e Pescoço, FMRP, USP, Ribeirão Preto, SP, Brazil

**Keywords:** aging, balance, muscle strength, older people, rehabilitation

## Abstract

**BACKGROUND:**

muscle strength and power are two factors affecting balance. The impact of muscle strength and power on postural control has not been fully explored among different age strata over sixty.

**OBJECTIVES:**

the aim of the present study was to assess the muscle strength and power of elderly women in different age groups and determine their correlation with postural control.

**METHOD:**

eighty women were divided into four groups: the young 18-30 age group (n=20); the 60-64 age group (n=20); the 65-69 age group (n=20); and the 70-74 age group (n=20). The participants underwent maximum strength (one repetition maximum or 1-RM) and muscle power tests to assess the knee extensor and flexor muscles at 40%, 70%, and 90% 1-RM intensity. The time required by participants to recover their balance after disturbing their base of support was also assessed.

**RESULTS:**

the elderly women in the 60-64, 65-69, and 70-74 age groups exhibited similar muscle strength, power, and postural control (p>0.05); however, these values were lower than those of the young group (p<0.05) as expected. There was a correlation between muscle strength and power and the postural control performance (p<0.05).

**CONCLUSION:**

despite the age difference, elderly women aged 60 to 74 years exhibited similar abilities to generate strength and power with their lower limbs, and this ability could be one factor that explains the similar postural control shown by these women.

## Introduction

The literature on gerontology clearly demonstrates that the elderly exhibit poorer balance compared to young individuals^1^^,^ 2. Nevertheless, a few studies have evaluated postural control throughout the aging process by analyzing the balance in elderly individuals from different age groups. The studies that sought to investigate this subject have reported contradictory results^3^^-^ 8. Some studies indicate that postural control performance differs between elderly populations from different age groups^4^^,^ 5 ^,^ 7, while other studies indicate that such behavior is similar among elderly individuals in different age strata^6^^,^ 8. Additionally, the previous studies analyzed a wide age range (10 years)^3^^-^ 8. However, Hofer and Sliwinski^9^ suggested that analysis of narrow age-cohort sample may be more suitable for deriving inferences regarding the interdependence of aging-related change. Several studies that investigated postural control described the elderly as all individuals older than 65 years^1^^,^ 2 ^,^ 10 and did not take into account possible differences in motor performance that could occur due to a small age difference (e.g. 5 years). Therefore, the question remains: do elderly individuals in different age strata actually exhibit similar postural control to support their classification into a single age group? Analysis of postural control in elderly individuals of small age ranges (5 years) and under different situations of balance maintenance could provide important information about the behavior of the postural control system throughout the aging process.

The biomechanical constraints associated with aging are known to exhibit a strong correlation with the decline in postural control in the elderly^11^. More specifically, elderly individuals are less able to generate muscle strength and power, and this inability appears to increase postural instability and the tendency to fall^12^^-^ 14. Several studies demonstrated a greater decrease in muscle power than in muscle strength in the elderly population^15^^,^ 16. In addition, muscle power compared to muscle strength was shown to be a better predictor of functional ability and susceptibility to falls in the elderly^12^^-^ 14 ^,^ 17.

To maintain balance after an external disturbance, a rapid generation of muscle force (muscle power) must be performed to react quickly to threats in order to stabilize the center of mass^17^. Johnson and Woollacott^18^ revealed that power-trained athletes presented more effective responses to recovery from a horizontal platform perturbation than endurance-trained athletes. In a study by Bean et al.^12^, the authors identified a correlation between the muscle power produced by the lower limbs and a set of functional tests. The results indicated a significant correlation between the functional tests and the muscle power generated at an intensity of 70% of one repetition maximum (1-RM)^12^. Puthoff and Nielsen^13^ identified the strongest correlation between muscle power and clinical tests at an intensity of 90% of 1-RM^13^. In a study by Orr et al.^19^, elderly men and women were subjected to 20 sessions of muscle power training at different relative intensities (20%, 50%, and 80% of 1-RM), and the results indicated that low-intensity (20% of 1-RM) power training promoted the greatest benefit to postural balance.

The conclusions of previous studies^12^^,^ 13 ^,^ 17 ^-^ 19 indicate correlation between muscle power and postural balance, nevertheless there is no agreement in the literature with respect to the relative power intensity needed to recover balance after an external disturbance. Although several studies have demonstrated that the elderly exhibit decreased muscle strength and power compared to young adults^3^^,^ 15 ^,^ 20, few studies have sought to investigate whether such abilities differ among elderly individuals in different age groups^3^^,^ 21. In a recent study, Pedrero-Chamizo et al.^21^ examined 2,412 women older than 65 years and found that the oldest-old exhibited lower body strength compared to young-old women. Conversely, the results of the study by El Haber et al.^3^ indicated that the isometric strength of the knee extensor muscles was similar between elderly women in the 61-70 age group and the 71-82 age groups.

These data demonstrate the scarcity of studies on the influence of age on the motor performance of elderly women of different ages, as well as the lack of information concerning the ability of these women to generate muscle strength and, especially, muscle power. Such information could be useful in the elaboration of exercise programs designed to improve muscle power at the specific intensity that best achieves the maintenance of balance and the prevention of falls in the elderly population.

Therefore, the aim of the present study was to analyze the ability of elderly individuals of different ages to produce muscle power at specific relative intensities and to analyze the correlation of muscle performance with postural control. Differences were expected to be found between the 60-64 age group and the adjacent 5-year age range with respect to postural control and muscle power.

## Method

### Participants

Eighty healthy women participated in the study. The participants were recruited from community and were divided into four groups according to their age strata: young group (n=20; aged 18 to 30 years), 60-64 group (n=20; aged 60 to 64 years); 65-69 group (n=20; aged 65 to 69 years); and the 70-74 group (n=20; aged 70 to 74 years). All of the participants signed an informed consent form approved by the local ethics committee (no. 459/2009, Research Ethics Committee of HC and Faculdade de Medicina de Ribeirão Preto, Universidade de São Paulo (FMRP-USP), Ribeirão Preto, SP, Brazil). The exclusion criteria included the following: bone fracture or injury in the lower limbs in the previous six months, vestibular disorder, neuropathy, neurological disorders or any other musculoskeletal problem affecting the participants' ability to maintain an upright posture or to sit and rise from a chair.

### Procedures

Data were collected at the Laboratory of Balance Assessment and Rehabilitation (LARE). After the procedures were explained, the participants were asked to answer three questionnaires: 1) Brazilian Multidimensional Functional Assessment Questionnaire (BOMFAQ)^22^; 2) Modified Baecke Questionnaire for Older Adults^23^; and 3) Falls questionnaire, used to assess the incidence of falls, fear of falling, and the circumstances and consequences of falls. A fall was defined as "unintentionally coming to rest on the ground, the floor or other lower level; excludes coming to rest against furniture, a wall or other structure"^24^. The participants were considered physically active when they reported performing at least three 30-minute sessions of physical activity per week. These questionnaires were used to obtain better control of the sociodemographic, physical, and functional characteristics of the elderly participants, thus were not used for the young group.

The strength and power of the knee extensor and flexor muscles were measured using a flexor/extensor chair. During the assessment of muscle strength and power, the back of the chair was adjusted so that the participants were able to maintain a hip angle of 90º and the equipment and knee axes coincided. The leg support was placed above the ankle joint. The knee extension and flexion movements were performed unilaterally, and the values obtained for the right lower limb were used for the purpose of comparison, as the right side was the dominant side reported by 95% of the participants.

The concentric strength of the knee extensor and flexor muscles was measured using the 1-RM test^25^. The concentric power of the knee extensor and flexor muscles was measured at 40%, 70%, and 90% of 1-RM. The participants were instructed to perform the knee extension or flexion movement as fast as they were able. Power was calculated as the product of torque and angular velocity at each instant in time. The highest power value observed (peak power) was used to describe the muscle power generated by the knee extensors and flexors at each investigated relative intensity. Each participant performed two attempts at each investigated intensity, and the arithmetic mean of these two power values was used in the statistical analysis.

The performance of the postural control system was measured using a force plate (Synapsys Posturography System - Synapsys S/A, Marseille, France) with 100 Hz sampling frequency. The participants stood barefoot on the platform, placing their feet approximately 10 cm apart according to the manufacturer's instructions. Once a trial began, the platform moved translationally in the anterior-posterior (AP) and mediolateral (ML) directions, disrupting the participants' balance. To accomplish AP translation, the platform moved forward at a speed of 3.0 cm/s and an amplitude of 6.0 cm; after an 8-second interval, it moved backwards at the same speed and amplitude. To accomplish ML translation, the parameters were the same, but the platform moved first to the right and subsequently to the left. Each attempt included six translations and lasted a total of 51.2 seconds. Two trials were conducted for direction (AP and ML, respectively), the first trial with the participant's eyes open and the second trial with the participant's eyes closed, for a total of four attempts. The performance of postural control was the time's measured elapsed between the onset of the disturbance and the participant's recovery of balance. Balance is recovered when the center of pressure (CoP) returns to a position between -2 and +2 millimeters from the position held prior to the onset of the balance disruption.

### Statistical analysis

The Shapiro-Wilk's test for normality and the Levene test for homogeneity of variance indicated that the variables exhibited normality and homogeneity of variance. A pilot study was conducted to calculate the sample size. We chose the variable Recovery Time in the AP direction with eyes open to perform the sample size calculation with the following assumptions: α=0.5; β=0.8; 15% of maximum difference; and standard deviation = 0.8. The sample size calculation indicated 14 subjects. To investigate the similarity of the anthropometric and sociodemographic data, six analyses of variance (ANOVA) were performed using the group as the factor and the age, body mass, height, body mass index (BMI), functional ability, and physical fitness acting as the dependent variables. To analyze muscle strength, muscle power, and postural control performance, multivariate analyses (MANOVA) were performed.

To analyze the correlation between muscle strength and power and postural control performance, Pearson's correlation tests were performed. Chi-square (X^2^) tests were performed to compare the occurrence of falls, fear of falling down, and practice of physical activity between groups (60-64 vs. 65-69 vs. 70-74 age groups).

Whenever needed, univariate analyses and post hoc tests (Tukey) were used. All of the statistical analyses were performed using the SPSS software (SPSS for Windows, V16.0 - SPSS Inc., USA), and the level of significance was set at 0.05.

## Results

### Characteristics of groups

Table 1 describes the age, anthropometric, and sociodemographic data of the participants as a function of age. Univariate analyses demonstrated significant differences in body mass [F(3,76)=5.36, p<0.05], height [F(3,76)=8.89, p<0.05] and BMI [F(3,76)=12.84, p<0.05] between the groups. Post-hoc tests indicated that the participants in the young group exhibited lower body mass compared to those in the 65-69 and 70-74 age groups. In addition, the participants in the young group exhibited lower BMIs and greater height compared to the other groups. With respect to age, ANOVA identified a significant difference among all groups [F(3,76)=350.60, p<0.05]. The proportion of physically active women (X^2^=1.30, p>0.05) and of those who reported falls (X^2^=0.41, p>0.05) or fear of falling (X^2^=3.60 p>0.05) were similar among all three groups of elderly participants.

**Table 1:** Mean±standard deviation (SD) of age, anthropometric data (body mass, height, and BMI) and sociodemographic characteristics (functional ability, physical fitness, practice of physical activity, and falls) for participants as a function of age groups.

	Groupyoung	Group60-64 years	Group65-69 years	Group70-74 years
Age (years)	22.5±2.7^^^	62.8±1.3^^^	66.7±1.4^^^	73.2±1.1^^^
Body mass (kg)	57.1±8.2	64.9±10.5	68.8±11.4^*^	67.4±9.3^*^
Height (cm)	161.0±6.0	154.0±6.0^*^	154.0±6.0^*^	153.0±4.0^*^
BMI (kg/m^2^)	22.0±3.3	27.1±4.0^*^	29.0±4.8^*^	28.7±4.1^*^
Functional ability (BOMFAQ)	_	1.8±0.9	1.6±0.5	1.5±0.8
Physical fitness (Baecke)	_	16.4±4.0	16.3±3.8	18.8±2.1
Subjects who performed physical activity (%)	_	75.0	70.0	85.0
Subjects who fell (%)	_	40.0	35.0	45.0
Subjects with fear of falling (%)	_	50	65	35

* Significantly different from the young group, P<0.05; ^Significantly different from all other groups, P<0.05.

### Muscle strength and power

**Figure 1:**
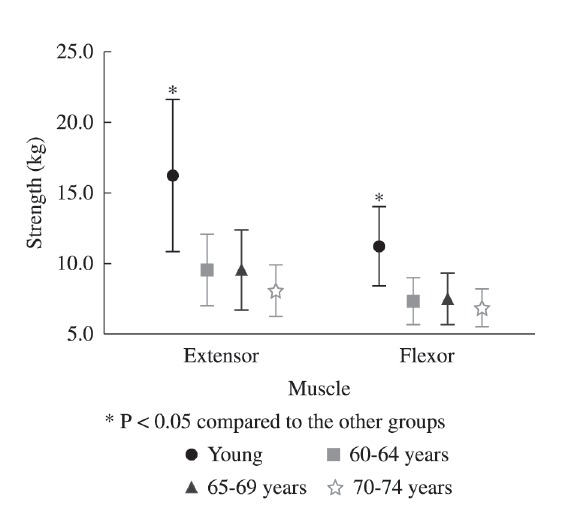
Mean and standard deviation of muscle strength of the knee extensors and flexors for the different age groups (young, 60-64, 65-69, and 70-74 years old).

As a whole, the results demonstrated that the elderly women (60-64, 65-69, and 70-74 age groups) exhibited similar strength and power, which were lower compared to the young women. Figure 1 depicts the strength of the knee flexor and extensor muscles of the four investigated groups. MANOVA indicated a significant difference for the group factor [Wilks' Lambda=0.48, F(6,150)=10.86, p<0.05]. Univariate analyses indicated significant differences in knee extensor [F(3,76)=22.76, p<0.05] and flexor muscles [F(3,76)=20.50, p<0.05]. Post-hoc tests demonstrated that the young women exhibited greater strength in both the extensor and flexor muscles compared to all three groups of elderly women (60-64, 65-69, and 70-74 age groups).

**Figure 2:**
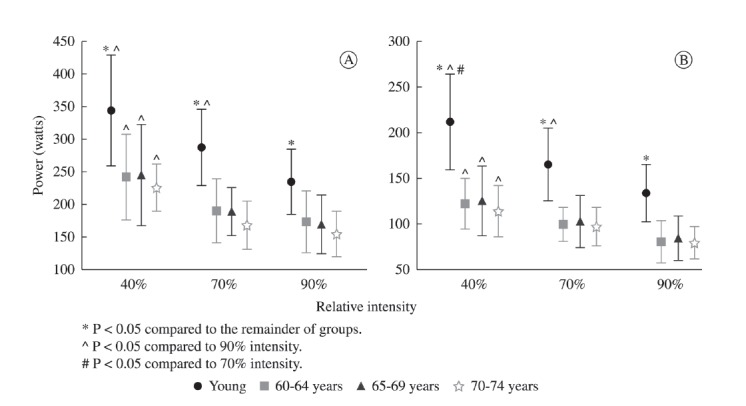
Mean and standard deviation of muscle power of the knee extensors (A), and knee flexors (B) at the relative intensities of 40%, 70%, and 90% 1-RM among the different age groups (young, 60-64, 65-69, and 70-74 years old).

Figure 2 depicts the power values produced by the knee extensor (a) and flexor (b) muscles at intensities of 40%, 70%, and 90% of 1-RM in the four investigated groups. MANOVA indicated significant differences for group [Wilks' Lambda=0.42, F(6,150)=3.22, p<0.05], intensity [Wilks' Lambda=0.12, F(4,73)=124.90, p<0.05], and group-intensity interaction [Wilks' Lambda=0.52, F(12,193)=4.41, p<0.05].

With respect to the power of the knee extensor muscles (Figure 2A), univariate analyses revealed significant differences among the groups [F(3,76)=19.59, p<0.05], intensities [F(2,152)=146.19, p<0.05] and group-intensity interaction values [F(6,152)=2.49, p<0.05]. Post-hoc tests indicated that the young group exhibited greater power compared to the remainder of the groups. These post-hoc tests also indicate that power at 40% intensity was greater than the power attained at 70% and 90% of 1-RM for all of the investigated groups. In the young group, the power at 70% of 1-RM was greater than the power produced at 90% of 1-RM.

Regarding the power of the knee flexor muscles (Figure 2B), univariate analyses indicated significant differences among the groups [F(3,76)=28.57, p<0.05], intensities [F(2,152)=313.76, p<0.05] and group-intensity interactions [F(6,152)=13.57, p<0.05]. Post-hoc tests indicated that the young group exhibited greater muscle power than the other three groups. In addition, the results revealed that the power attained at 40% of 1-RM was greater than the power at 90% of 1-RM in all of the investigated groups. The group of young women also exhibited significant differences between the intensities of 40% and 70% of 1-RM, as well as differences between 70% and 90% of 1-RM.

### Postural control

**Figure 3:**
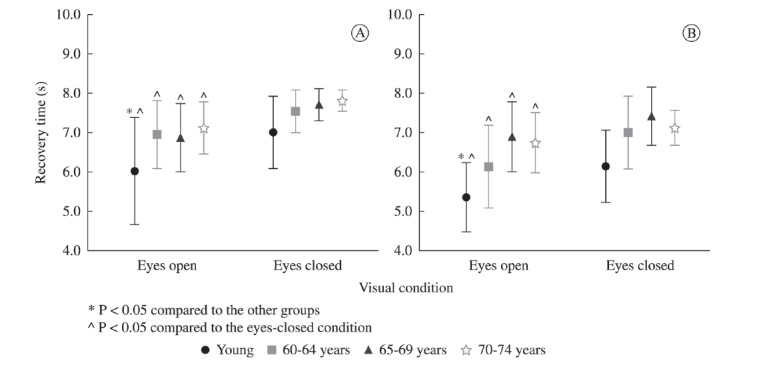
Mean and standard deviation of AP (A) and ML (B) recovery times with eyes open (EO) and eyes closed (EC) for all of the age groups (young, 60-64, 65-69, and 70-74 years old).

Figure 3 depicts the balance recovery time values after translation of the platform in the AP (a) and ML (b) directions for all four investigated groups. MANOVA identified significant differences for the group [Wilks' Lambda=0.58, F(6,150)=7.80, p<0.05] and vision [Wilks' Lambda=0.39, F(2,75)=58.6, p<0.05].

Univariate analyses indicated significant differences among the groups [F(3,76)=7.27, p<0.05] and vision [F(1,76)=77.49, p<0.05] in response to translation in the AP direction (Figure 3A). Post-hoc tests indicated that the young group exhibited shorter recovery times compared to the other three groups. In all groups, the recovery time increased when the participants performed the exercises with their eyes closed.

For translation in the ML direction (Figure 3B), univariate analyses also indicated significant differences among the groups [F(3,76)=15.3, p<0.05] and vision [F(1,76)=40.71, p<0.05]. Post-hoc tests indicated that the young group exhibited shorter recovery times compared to the other three groups. In all groups, the recovery time increased when exercises were performed with eyes closed.

Correlation between muscle strength and power and postural control

The correlation coefficient (r) between the measurements of muscle strength and power and the variables of postural control performance are described in Table 2. The results indicate that the strength of the knee extensor and flexor muscles and the power produced by the knee extensors at 70% and 90% of 1-RM correlate with the postural control performance. The results also reveal that the greater the strength of the knee extensor muscles, the shorter the balance recovery time in the AP direction with eyes open or closed and the shorter the balance recovery time in the ML direction with eyes open. In addition, the results indicate that the greater the strength of the knee flexor muscles, the shorter the balance recovery time in the ML direction with eyes open. Finally, the results indicate that the greater the power produced by the knee extensor muscles at 70% and 90% of 1-RM, the shorter the balance recovery time in the AP direction with the eyes open.

**Table 2:** Pearson's correlation (r) values between the measurements of muscle strength and power and the balance recovery time on the anterior-posterior (AP) and mediolateral (ML) directions with the eyes open (EO) or closed (EC).

Measurements	Recovery time (AP-EO)	Recovery time (AP-EC)	Recovery time (ML-EO)	Recovery time (ML-EC)
Knee extensorStrengthPower at 40% 1-RMPower at 70% 1-RMPower at 90% 1-RM	-.25*.20-.24*-.25*	-.28*-.01-.12-.17	-.35*.02-.10-.06	-.17.03-.09-.01
Knee flexorStrengthPower at 40% 1-RMPower at 70% 1-RMPower at 90% 1-RM	-.17-.02-.01-.10	-.16.10.12.01	-.30*.15.17.08	-.20.13.18.21

* P<0.05.

## Discussion

The investigation of muscle strength, muscle power, and postural control in shorter age intervals in elderly women allows us to understand the impact of the aging process on balance in the elderly, a relevant aspect considering that impaired balance increases the risk of falls.

The results revealed that the elderly participants in the age ranges of 60-64, 65-69, and 70-74 years exhibited similar postural control and had similar muscle performance. The present findings contradict prior studies indicating that postural control performance differs between elderly populations from different age groups^4^^,^ 5 ^,^ 7 and is in accordance with other previous studies that show no difference among elderly individuals in different age strata^6^^,^ 8. However, the previous studies^4^^-^ 8 analyzed a wide range of age stratification (10 years) and the present paper focused on smaller age strata (5 years). The preceding studies that investigated postural control generally described the elderly as all individuals older than 65 years or termed youngest old (up to 79 years) and oldest old (80 years and over)^1^^,^ 4 ^,^ 26 and did not take into account possible differences in motor performance that could occur due to a small age difference (5 years). The present results indicated that a range of 5 years of age seems to be insufficient to produce noticeable changes in the performance of the postural control system in healthy elderly women.

The efficiency of the postural control system depends on an intricate relationship between multiple sensory and motor components^11^^,^ 27. Consequently, deficits in any of these sensory-motor elements can be reflected in the postural control performance. The results of the present study confirm the fact that elderly women exhibit motor decline, which was evidenced by the lower muscle strength and power measured by the knee extensors and flexors compared to the young women, thereby corroborating the results of previous studies^3^^,^ 15 ^,^ 20 ^,^ 25 ^,^ 28. In addition, our findings indicate that between the ages of 60 and 74 years, the muscle performance was similar. Although the literature indicate that sarcopenia is related to age^29^, in the present age strata samples there were no differences among women in the age ranges of 60-64, 65-69, and 70-74 years. Moreover, the findings revealed that elderly women in the 60-64, 65-69, and 70-74 age groups exhibit similar muscle power regardless of the intensity relative to the maximum load. Assuming that different daily tasks (e.g. rising from a seat or walking) require production of muscle power at different relative intensities^12^^,^ 13, these results suggested that the elderly women of the three investigated groups (60-64, 65-69, and 70-74 years) are similarly adapted to meet such motor demands. These results represent a new finding within geriatrics and gerontology research; no other study to date has specifically assessed the relative power of the knee extensor and flexor muscles in elderly women of different ages.

With respect to muscle strength, previous studies have demonstrated that the strength of the lower limbs is similar among elderly women of different ages, which is confirmed by the present study^3^^,^ 30. It is worth stressing that, in the present study, most (>70%) of the participants in the three groups of elderly women were physically active. The practice of physical exercise might have contributed to the fact that the women in the higher age strata (65-69 and 70-74 years) exhibited levels of muscle strength and power similar to the ones in the lower age stratum (60-64 years)^31^.

Several studies reported that the muscle strength and power of the lower limbs correlate with postural control performance and with functional abilities^12^^-^ 14 ^,^ 16 ^,^ 19. The studies by Bean et al.^14^ and Orr et al.^19^, for example, demonstrate that the greater the muscle power of the knee and hip extensors, the better the postural balance. Therefore, because the elderly women of the three investigated groups (60-64, 65-69, and 70-74 years old) exhibited the same ability to generate muscle power, one might expect these groups to exhibit similarities in postural control performance.

By means of Pearson's correlation, the present study indicated that the muscle strength of the knee extensors and flexors and the muscle power of the knee extensor correlated with the postural control performance. With respect to the relative intensity in which power was generated (40%, 70%, and 90% of 1-RM), the power of the knee extensors at moderate (70% of 1-RM) and high (90% of 1-RM) intensity were correlated with balance maintenance. Similar to the findings by Bean et al.^12^, our results indicate that the power generated at a moderate relative intensity (70% of 1-RM) correlates better with balance compared to power generated at a low (40% of 1-RM) relative intensity. These results suggested that professionals should increase progressively the load in order to reach the muscle power necessary to recover to external postural disturbances.

Although the present study demonstrated that the muscle strength and power of the knee extensors and flexors correlated with the postural control performance of elderly women and thus corroborated previous studies^17^^,^ 19 ^,^ 32, such correlation must be interpreted carefully because the values of the correlation coefficient were low (lower than 0.35). In a thorough literature review, Orr^17^ found that the evidence for the correlation between muscle strength and power and postural balance is still limited, suggesting that many intrinsic and still unknown factors might be associated with variations in postural balance^17^.

These intrinsic factors may be related to anthropometric characteristics, sociodemographic conditions, lifestyles, functional abilities, physical fitness, and history of falls^1^^,^ 33 ^-^ 35. The present study found that the elderly women in different age groups exhibited similar functional abilities and levels of physical fitness. In addition, the number of women who reported falls or fears of falling and who were physically active was similar in all three investigated groups. However, other intrinsic aspects such as timing of muscle contraction, sequence of muscle activation, and intersegment coordination were not evaluated. Furthermore, it is important to emphasize that the elderly included in this study were healthy and active, but other variables such as comorbidities are playing an important role in normal aging.

In conclusion, the present study demonstrated that elderly individuals in the age range of 60-74 years exhibited similar postural control muscle performance. Moreover, our results indicate that the power of the knee extensors at moderate (70% of 1-RM) and high (90% of 1-RM) intensity were correlated with balance maintenance. These results suggested that professionals should increase progressively the load in order to reach the muscle power necessary to recover to external postural disturbances.
